# Improving the Quality of Children’s Mental Health Care with Progress Measures: A Mixed-Methods Study of PCIT Therapist Attitudes

**DOI:** 10.1007/s10488-021-01156-0

**Published:** 2021-08-07

**Authors:** Corinna C. Klein, B. Erika Luis Sanchez, Miya L. Barnett

**Affiliations:** 1 University of California, Santa Barbara, USA; 2 Department of Counseling, Clinical, & School Psychology, Santa Barbara, CA 93106-9490 USA

**Keywords:** Progress measures, Implementation, PCIT, Measure attitudes

## Abstract

Progress measures are an evidence-based technique for improving the quality of mental health care, however, clinicians rarely incorporate them into treatment. Research into how measure type impacts clinician preference has been recommended to help improve measure implementation. Parent–Child Interaction Therapy (PCIT) is an assessment-driven treatment that serves as an ideal intervention through which to investigate measure preferences given its routine use of two types of assessments, a behavioral observation (the Dyadic Parent–Child Interaction Coding System) and a parent-report measure (the Eyberg Child Behavior Inventory). This study investigated PCIT therapist attitudes towards progress measures used within PCIT and children’s mental health treatment generally. A mixed-method (QUAN + QUAL) study design examined PCIT therapist attitudes towards two types of progress measures and measures used in two contexts (PCIT and general practice). Multi-level modeling of a survey distributed to 324 PCIT therapists identified predictors of therapist attitudes towards measures, while qualitative interviews with 23 therapists expanded and clarified the rationale for differing perceptions. PCIT therapists reported more positive attitudes towards a behavioral observation measure, the DPICS, than a parent-report measure, the ECBI, and towards measures used in PCIT than in general practice. Clinician race/ethnicity was significantly related to measure-specific attitudes*.* Qualitative interviews highlighted how perceptions of measure reliability, type of data offered, ease of use, utility in guiding sessions and motivating clients, and embeddedness in treatment protocol impact therapist preferences. Efforts to implement progress monitoring should consider preferences for particular types of measures, as well as how therapists are trained to embed measures in treatment.

## Introduction

One in five youth in the United States experience a serious mental disorder in their lives (Merikangas et al., 2010). Without effective intervention, youth mental health problems have deleterious trajectories resulting in high personal and societal consequences, including increased medical costs, emergency room usage, and engagement in the criminal justice system (Rivenbark et al., 2018). The quality and effectiveness of children’s mental health services remains concerning, as 24% of youth who do receive services in community mental health centers actually get worse (Warren et al., 2010). A large naturalistic study of youth who received mental health services in community settings found that the problem severity of 55.7% did not change at all in treatment, and that the functioning of 14.7% of youth actually declined over the course of treatment (Smith & Jensen-Doss, 2017). This study found that younger youth were more likely to demonstrate deterioration in treatment than older youth (Smith & Jensen-Doss, 2017). In order to improve care, quality improvement strategies for youth mental health services that mitigate deterioration in treatment and ensure that youth are receiving effective services should be identified.

Implementation research and policy reform has addressed this issue by attempting to increase the use of evidence-based practices (EBPs) within community settings. Unfortunately, this approach has faced many clinician-level and system-level barriers, including high training and implementation costs, clinician aversion to adopting new treatment models, and low fidelity to treatment protocols (Beidas et al., 2015; Damschroder et al., 2009; Lyon et al., 2018). Community agencies see clients with greater diversity, stressors, and clinical complexity than the populations on which EBP efficacy was originally evaluated, limiting their utility for broader client populations (Ehrenreich-May et al., 2011; Southam-Gerow et al., 2012). These differences have been noted by agency leaders in community settings, who have reported that EBPs are often not suited to the clinical presentations, ages, or family compositions of their clientele (Rodriguez et al., 2018). Evidence-based assessments offer a complementary approach to quality improvement that circumvents many of these implementation and feasibility barriers. Evidence-based assessments, which are also called progress measures, can be applied across diagnoses, treatment settings, and theoretical orientations, allowing clinicians to maintain their theoretical orientations and precluding lengthy agency-wide trainings in new treatment models (Lyon, et al., 2018). Assessments create an individualized evidence base within clinical practice, allowing practitioners who may not attend to the wider clinical literature to see first-hand how effective their services are and to cater their clinical decisions to their clients’ progress and needs (Bickman et al., 2016). Assessments may be useful alternatives in instances frequently encountered in community agencies, in which client age, complexity, or comorbidity excludes them from meeting criteria for an available EBP (Park et al., 2018). They may also be used to supplement available EBPs to ensure progress and enhance treatment.

Monitoring patient progress by using standardized measures has been associated with improved mental health care, particularly for patients who are not responding to treatment or who are actively deteriorating (Bickman et al., 2011; Lambert & Shimokawa, 2011; Russel et al., 2018). Outcome measures are valued by both clinicians and clients, who demonstrate increased engagement in collaborative treatment planning and improved self-awareness (Solstad et al., 2019). Implementation of progress monitoring in routine care has been recommended as an important component of patient-focused treatment due to its ability to mitigate patient deterioration (Boswell et al., 2015; Goodyear et al., 2017). The American Psychological Association’s Presidential Task Force on Evidence-Based Practice identified progress monitoring as an important element of clinical practice (APA, 2006). Despite evidence supporting the use of measures and assessments as a component of effective treatment, a small minority of clinicians currently use them (Jensen-Doss et al., 2018a, 2018b). Researchers have found that clinicians report positive attitudes towards measures, but still do not implement them in routine practice (Jensen-Doss, et al., 2018a, 2018b). Therapists rely significantly more on clinical intuition than on validated assessments in evaluating patient progress, despite evidence that therapist judgement is often an inaccurate barometer of client progress (Garland, et al., 2003; Hatfield, et al., 2009). These findings together suggest that adoption of routine progress monitoring may face many of the same challenges that implementation of EBPs faces.

Since progress measures have the potential to enhance mental health treatment and improve patient outcomes by empowering patients, providing feedback to clinicians, and promoting informed treatment planning, an investigation of the discrepancy between clinician attitudes towards and use of progress measures is necessary. Researchers have recommended an investigation of this discrepancy, as well as research into specific factors that may influence clinician use of progress measures (Boswell et al., 2015). Additionally, recent findings that clinicians prefer particular forms of progress measures suggest that some measures may be more acceptable to clinicians than others (Jensen–Doss et al., 2018a, 2018b). These findings on progress measures are consistent with studies demonstrating that therapist attitudes towards EBPs vary based on practice characteristics (Barnett et al., 2017; Reding et al., 2014). Research into which measures clinicians prefer will allow agencies and policy makers to cater their quality improvement efforts by implementing measures that clinicians find acceptable and are more likely to use. Implementation science, an area of research aimed at increasing access to evidence-based and effective services in community settings, has repeatedly highlighted the importance of investigating the attitudes of services providers towards evidence-based practices (Aarons, 2004, 2005; Nelson et al., 2006). In order to understand low utilization of progress measures, research should investigate therapist attitudes and preferences (Rye et al., 2019), focusing specifically on whether there are measures therapists prefer to use (Jensen-Doss et al., 2020).

Widespread dissemination of EBPs that embed progress monitoring in their protocols allows for the investigation of therapist attitudes towards progress monitoring when it is explicitly linked to treatment. Although therapist attitudes towards these measures may not be entirely separable from the practices in which they are embedded, they offer an initial means of studying the preferences of therapists who have substantial experience with multiple types of measures. PCIT provides an ideal EBP to study with regards to therapist attitudes towards progress measures for two important reasons. First, it is an assessment-driven treatment, with weekly assessments determining when therapists teach parents new skills and when the family graduates from treatment (Bahl et al., 1999). Secondly, PCIT uses standardized progress measures with unique characteristics, including parent report measures (i.e. the Eyberg Child Behavior Inventory; ECBI) and behavior observations (i.e., the Dyadic Parent–Child Interaction Coding System; DPICS). The DPICS is used to evaluate changes in parent and child behaviors in PCIT. A longer version of the behavior observation is conducted at pre- and post-treatment, while a shorter version is conducted during most treatment sessions (Eyberg et al., 2013). The Eyberg-Child Behavior Inventory (ECBI) is a brief, parent report paper and pencil measure of child behavior problems (Eyberg & Pincus, 1999). The ECBI includes two scores: the Intensity Score, calculated based on responses to 36 items on a 7-point scale, indicates the intensity of the child’s difficult behaviors, while the Problem Score, calculated based on 36 yes or no questions, indicates how problematic the caregiver finds those behaviors. These assessments help clinicians evaluate child behaviors and parent proficiency with the skills, so that treatment is ideally not completed before parents reach a high level of skill proficiency and the child’s behaviors are subclinical, which may help maintain long-term treatment gains (Eyberg et al., 2014).

PCIT has over 40 years of research demonstrating that it is an effective intervention that improves the parent–child relationship, increases parenting skills, decreases child conduct problems, and reduces physical maltreatment, with multiple studies demonstrating that PCIT outperforms treatment-as-usual in community settings (Lieneman et al., 2017). The treatment is divided into two phases, Child-Directed Interaction (CDI) and Parent-Directed Interaction (PDI), each of which entails a session during which caregivers are taught parenting skills in a didactic format and then receive in-vivo coaching on skill usage (Eyberg, S. M. Funderburk & B, 2011). Given these outcomes, major county and statewide initiatives have supported the implementation of PCIT to improve outcomes for young children with conduct problems and families at-risk for physical maltreatment (Beveridge et al., 2015; Timmer et al., 2016). This widespread dissemination allows for a national study of clinician attitudes towards multiple types of progress measures in community-based care.

### Current Study

This study used a mixed-methods design, which has been recommended for implementation science due to its ability to provide insight into the gap between research findings (e.g. the ability of progress measures to improve treatment) and clinical practice (e.g. low use of progress measures by clinicians) by merging multiple types of data (Palinkas et al., 2011). In this study, mixed-methods were used to investigate the attitudes of PCIT therapists towards two distinct types of measures used within PCIT (the DPICS and the ECBI) and standardized assessments used in routine practice. The study seeks to answer two questions: 1) Do PCIT therapists prefer the DPICS, a behavior observation measure, or the ECBI, a parent report measure? 2) Do PCIT therapists prefer measures used within PCIT or those used in general practice? The study included quantitative data from a larger survey distributed to PCIT therapists, as well as qualitative data gathered through interviews with a subset of therapists who completed the survey.

## Method

### Participants

Participants were recruited from email listservs for PCIT therapists and were eligible for the study if they had seen a PCIT client in the last 2 months. From the 411 survey respondents, 26 did not meet study criteria of having seen a PCIT client in the last two months, and 61 did not complete any of the survey beyond demographic information (15%), and were therefore excluded from analyses. This yielded a final quantitative sample size of 324.

Survey participants were predominately female (91.7%), non-Hispanic white (74.2%), and Master’s level clinicians (69.7%) with an average of 8.88 (*SD* = 6.99) years of experience providing mental health services. Qualitative interviews were conducted with a subset of 23 mental health professionals who had completed the survey (87% female, 69.65% non-Hispanic white). Interviewees were selected by criterion-i sampling, a sampling strategy that purposefully selects participants based on predetermined criteria of interest, in order to identify cases for in-depth qualitative follow-up (Palinkas et al., 2015). In this study, criteria sampled for were working in community settings and providing services in multiple languages. In accordance with the aims of implementation science, which seeks to evaluate the community provision of evidence-based practices, interviews were only conducted with clinicians providing services in community-based settings. Additionally, due to the dearth of information on cultural considerations as they relate to progress monitoring (Rodriguez et al., 2019), therapists who provide services in more than one language were oversampled. Twelve participants reported providing services in both English and another language (52.1%), the majority of whom delivered services in both English and Spanish (*n* = 9). Demographics for the survey and interview sample are included in Table 1.

**Table 1:** Demographic and professional characteristics of survey and survey + interview sample

	Survey sample	Interview sample
Demographics		
Age, *M (SD;* range) n = 323	36.92 (8.52; 22–71)	37.30 (6.68; 29–59)
N (%) Female n = 324	297 (91.7%)	20 (87%)
Race/ Ethnicity n = 322	*n* (%)	
Non-Hispanic White	239 (74.2%)	16 (69.6%)
Latinx/Hispanic	46 (14.3%)	7 (30%)
Other Ethnicity	37 (11.5%)	0 (0%)
Race n = 313	*n* (%)	
White	266 (85.0%)	22 (95.7%) (1 missing)
Black/African American	8 (2.6%)	0
Asian American/Pacific Islander	10 (3.2%)	0
American Indian/Alaska native	2 (.6%)	0
Multiracial	11 (3.5%)	0
Not Listed	16 (5.1%)	0
Professional Characteristics		
Language of service provision	*n* (%)	
English only	251 (77.5%)	11 (47.8%)
Spanish	61 (18.8)	9 (39.1%)
Other	12 (3.7%)	3 (13%)
Professional Discipline n = 323	*n* (%)	
Clinical Psychology	109 (33.7%)	8 (34.8%)
Social Work	74 (22.9%)	5 (21.7%)
Counseling	68 (21.1%)	3 (13%)
Marriage Family Therapy	64 (19.8%)	5 (21.7%)
Not Listed	8 (2.4%)	2 (8.7%)
Highest Degree Obtained	*n* (%)	
Master’s degree	225 (69.7)	16 (69.6%)
Doctoral degree	87 (27.5)	7 (30.4%)
Other	10 (3.4%)	0
Theoretical orientation n = 322	*n* (%)	
Cognitive Behavioral or Behavioral	219 (68.0%)	13 (56.5%)
Family Systems	51 (15.8%)	1 (4.3%)
Psychodynamic	9 (2.8%)	2 (8.7%)
Humanistic	9 (2.8%)	1 (4.3%)
Other (e.g. eclectic, attachment-based, combination of above)	34 (10.5%)	6 (26.1%)
N (%) who are licensed clinicians n = 323	256 (79.3%)	22 (95.7%)
N (%) PCIT Certified Therapist	228 (70.4%)	20 (87%)
N (%) PCIT Certified Trainers	99 (30.6%)	11 (47.8%)
Years Trained in PCIT *M (SD;* range) n = 323	5.25 (4.96; 0–37)	5.35 (3.43; 1–13)
Years as therapist *M (SD;* range) n = 324	8.88 (6.99; 0–45)	8.30 (4.24; 2–22)
Current caseload characteristics		
Current PCIT caseload *M (SD;* range) n = 323	5.75 (4.99; 0–30)	6.26 (3.85; 1–13)
Current Total caseload *M (SD;* range) n = 320	20.84 (18.64; 1–100)	19.17 (12.80; 3–50)

### Procedure

Data for both the quantitative and qualitative portions of this study was collected as part of a larger study investigating the experiences of clinicians with delivering PCIT. The study received an exemption from the Institutional Review Board at the University of California Santa Barbara. Participants were recruited through two PCIT listservs managed by PCIT International and PCIT Davis. They first completed a screening question asking whether they had seen a PCIT client in the past two months. Participants received a $20 gift card for completing the survey, which was not contingent on how many responses they provided.

The survey asked whether participants would be willing to participate in a supplemental interview. Interview participants received an additional $40 gift card for participation in an hour-long interview. Interviews were conducted by four graduate student researchers with expertise in providing PCIT and implementation science.

Of the 324 who completed the survey, 180 participants indicated that they would be willing to be interviewed (56%). Ten pilot interviews were conducted with early survey respondents to refine the interview protocol. Using the criterion sampling described above, the remaining eligible survey respondents who were willing to participate in interviews were randomized using an online random sample generator. Invitations to interview were sent via email to 43 participants, with one reminder follow-up email to those who did not respond to the initial invitation. Twenty participants did not respond, declined to interview, or did not show up to their interview (47%). It has been found that meaning saturation, the point at which both themes have been identified and depth of understanding has been conveyed, occurs between 16–24 interviews (Hennink et al., 2017). The research team met weekly to discuss interview content and emerging themes. Interviewers reviewed the subject matter of the previous week’s interviews along with non-interviewing members of the research team, including discussion of notable topics, anecdotes, and general content. A research member transcribed notes of the discussion in order to help identify recurrent content and emerging patterns. Interview content became redundant after 23 interviews, indicating that saturation had been reached, at which point interviews were completed.

### Measures

#### Therapist Characteristics

Participants completed the Therapist Background Questionnaire (Brookman-Frazee et al., 2012), which includes questions about their personal and professional characteristics. Demographic variables included age, gender, race (“What is your race: White; Black/African American; American Indian or Alaska Native; Asian American/Pacific Islander; Multiracial; Other (please specify),” and ethnicity (are you Hispanic/Latina/o/x?). A race/ethnicity variable was then created by combining and recoding the race and ethnicity variables, such that Latino/Hispanic included anyone identifying as such on the ethnicity variable, non-Hispanic white included anyone identifying as white (race variable) and non-Latino/Hispanic (ethnicity variable), and Other Minority included anyone identifying as Black/African American; American Indian or Alaska Native; Asian American/Pacific Islander; Multiracial; Other (race). Although important information is lost when aggregating racial and ethnic identities, this recoding allowed the inclusion of race/ethnicity in the quantitative model given limited diversity in our sample.

Professional background variables included licensure status, mental health discipline, highest degree obtained, and language of service provision. Workload variables included number of hours in direct service per week and the number of clients in caseload. Given the focus on PCIT in this study, additional variables included: PCIT certification status (e.g., in training, certified, certified as a trainer), years of experience with PCIT, and number of PCIT clients on caseload.

#### Attitudes towards Standardized Assessment Scales – Monitoring and Feedback Version (ASA-MF)

The ASA-MF **(**Jensen-Doss, et al., 2018a, 2018b) is an 18-item measure rated on a five-point scale from “strongly agree” (5) to “strongly disagree” (1) evaluating clinician attitudes towards progress measures. It contains three subscales: Clinical Utility (8 items), Treatment Planning (5 items), and Practicality (5 items). Clinicians completed the practicality scale (5 items) addressing their non-PCIT clients to evaluate attitudes towards use of progress measures in their routine practice. The scale had good internal consistency in this sample (α = 0.82).

#### Attitudes towards the DPICS and ECBI

To assess provider attitudes towards the DPICS and ECBI, the practicality subscale of the ASA-MF was modified, such that each item referred to “behavioral observation measures (e.g. DPICS)” or “parent report measures (e.g. ECBI)” rather than “standardized assessment.” Only one scale of the ASA-MF was repeated for the DPICS and ECBI to decrease response fatigue. The practicality subscale was selected because attitudes towards practicality were found to be the strongest and only independent predictor of use in a previous study by the developer of the measure (Jensen-Doss & Hawley, 2010; Jensen-Doss et al., 2018a, 2018b). The scale had adequate internal consistency in this sample for the behavioral observation measure (﻿α = 0.76) and good internal consistency for the parent report measure (﻿α = 0.85).

#### Semi-Structured Interview Guides

Therapists were asked a series of questions related to their attitudes towards progress monitoring generally (e.g., “How do you monitor progress with your non-PCIT clients?, “Do you encounter any barriers to using progress measures?”), as well as the DPICS (e.g. “What have you found to be helpful about using the DPICS?,”) and the ECBI (e.g. “What have you found to be helpful about using the ECBI?,”). The interview included questions comparing progress measures used within PCIT to progress monitoring in general practice (e.g. “how is using the DPICS and ECBI in PCIT different from using measures with other clients?”). The interview followed a funnel approach with broad inquiries followed by more specific follow-up questions aimed at clarifying and eliciting more detail (Spradley, 1979).

### Data Analysis

#### Mixed-Methods Design

This study used a QUAN + QUAL approach with simultaneous data collection and equal weighting of data in analyses (Palinkas et al., 2011). The quantitative data was used to compare therapist attitudes towards different types of progress measures. The qualitative data provided an important means of triangulation by clarifying and elucidating quantitative findings through therapist narrative, and expanded on the quantitative data by providing reasons for provider preferences.

#### Quantitative Data Analysis

Data were screened for invalid responses, with no invalid response patterns identified. No variable was missing more than 3.4% of its values. According to Little’s (1988) MCAR test, data were missing completely at random (﻿χ2 = 28.774, df = 22, *p* = 0.151). Therapist attitude scores on the ASA-MF practicality subscale for each measure (behavioral observation, parent-report, and general standardized assessment) were predicted using planned comparisons after confirming that all assumptions for a multilevel model were met. Attitudes towards the DPICS (*M* = 4.39*, SD* = 0.55*)* ranged from 2.20 to 5.00. Attitudes towards the ECBI (*M* = 4.24*, SD* = 0.65*)* ranged from 2.00 to 5.00, and attitudes towards standardized assessments in general practice (*M* = 3.83*, SD* = 0.69*)* ranged from 1.40 to 5.00. A multi-level model with random intercepts using SPSS v. 26 was conducted to account for the non-independence of attitude scores towards types of measures (Level 1) nested within therapists (Level 2), using listwise deletion to account for missing data, as recommended by Jakobsen et al. (2017).

The Level 1 outcome being predicted was the measure-specific attitude rating from the ASA-MF Practicality subscale. The ECBI was selected to be the reference group because as a paper-and-pencil measure, it is the most similar to typical standardized progress measures, which allowed for comparison to the DPICS in PCIT and to measures in general practice. Level 2 predictors included the following individual therapist characteristics: race/ethnicity (Latinx/Hispanic, Non-Hispanic White, Other Minority), Mental Health Discipline (Clinical Psychology, Social Work, Marriage Family Therapy, Counseling, Other), Education (Ph.D, M.A., less than M.A.), Age, Years as therapist, Licensure, and language of service provision (English only or English and another language). Therapists who provided services in another language (*n* = 73) provided them primarily in Spanish (n = 61, 18.8%), but also included Polish, German, American Sign Language, Portuguese, Vietnamese, Russian, Japanese, and Chamorro (*n* = 12, 3.7%). Type of measure (DPICS, ECBI, standardized assessment in general practice) was the primary predictor, with therapist demographic and professional characteristics entered as co-variates. Predictors and co-variates were selected based on previous research on predictors of both attitudes towards and use of progress measures (Edbrooke-Childs et al., 2017; Jensen-Doss et al., 2018a, 2018b; Sale et al., 2020). Although theoretical orientation has been found to predict attitudes, the present sample was not diverse enough in orientation for the inclusion of that variable to be informative (Jensen-Doss, et al., 2018a, 2018b).

#### Qualitative Data Analysis

Interviews were transcribed and audited by members of the research team. Thematic analysis of co-occurring codes was conducted using NVivo v 12 (Braun & Clarke, 2006). The authorship team developed a preliminary codebook of a priori codes based on the interview guide and then added emergent codes through an initial phase of coding. The coding team, which included undergraduate research assistants and graduate student researchers met regularly to review emergent codes, revise a priori codes, and resolve coding discrepancies. Through this iterative consensus process, a final codebook was developed with definitions of each code agreed upon by all members of the research team. Once the codebook was finalized, undergraduate coders coded all 23 interviews, with 50% of them coded by an advanced coder (1^st^ or 2^nd^ author) who met at intervals with the coding team to review any coding discrepancies and resolve them through dialogue and consultation with the entire research team as necessary. Following coding, thematic analysis involved analyzing quotes that emerged within co-occurrence of codes (e.g. DPICS + Clinical Utility) to produce themes. Thematic analysis was conducted by the coding team who met to investigate quotes, develop themes, and perform textual analysis. Themes were finalized collaboratively by the research team.

#### Integration of Quantitative and Qualitative Findings

The functions of this mixed-methods design were (1) Convergence – triangulating results to see if both yielded similar conclusions, (2) Complementarity – elaborating on and deepening understanding of quantitative data with qualitative narratives, and (3) Expansion – illuminating and further explicating quantitative results with qualitative data (Palinkas et al., 2011).

## Results

### Quantitative Results

Measure type was a significant predictor of therapist attitudes, *F* (2, 617.30) = 145.37, *p* < 0.001. Therapists endorsed more positive attitudes towards the DPICS (*EMM* = 4.34*, SE* = 0.08), *B* = 0.16, *p* < 0.001, and less positive attitudes towards standardized assessments used in general practice (*EMM* = 3.78*, SE* = 0.08), *B* = -0.40, *p* < 0.001, as compared to the ECBI (*EMM* = 4.18*, SE* = 0.08).

Race/ethnicity, *F* (2, 303.27) = 5.66, *p* = 0.004, and years as therapist, *F* (1, 303.43) = 5.88, *p* = 0.016, were significant predictors of measure-specific attitudes. Therapists who identified as Latinx or Hispanic (*EMM* = 3.86*, SE* = 0.11) reported less positive attitudes towards measures than therapists who identified as Non-Hispanic White (*EMM* = 4.16*, SE* = 0.09), *B* = -0.30, *p* = 0.008. More years of practice as a therapist predicted more positive attitudes towards measures, *B* = 0.02, *p* = 0.016. Although discipline, *F* (4, 301.563) = 2.13, *p* = 0.077, was not a significant predictor of measure-specific attitudes overall, therapists with degrees in Marriage and Family therapy (*EMM* = 3.95*, SE* = 0.11), *B* = -0.34, *p* = 0.005 and in Counseling (*EMM* = 4.02*, SE* = 0.11), *B* = -0.28, *p* = 0.021 reported less positive attitudes than those with degrees in Clinical Psychology (*EMM* = 4.29*, SE* = 0.08). Education, age, years of therapy practice, language of service provision, and licensure were not significantly related to measure-specific attitudes. Predictors for the model can be found in Table 2.

**Table 2:** Predictors of therapist attitudes

	*F*	Estimate
Intercept	509.48***	4.42***
Attitudes		
ECBI	145.37***	
DPICS		0.16***
Assessment in general practice		− 0.40***
Race and Ethnicity (Non-Hispanic White)	5.66**	
Latinx/Hispanic	−	− 0.30**
Other Ethnicity		0.12
Discipline (Clinical Psychology)	2.13	
Social Work		− 0.22
Marriage and Family Therapy		− 0.34**
Counseling		− 0.28*
Other		− 0.12
Education (Doctorate)	0.73	
Masters		0.14
BA or AA (*n* = 1)		0.08
Age	0.50	0.00
Years as Therapist	5.88*	0.02*
Licensure (not licensed)	1.73	
Licensed		− 0.11
Language (Only English)	.04	
All other languages		− 0.02

﻿*p < .05, **p < .01, ***p < .001

### Qualitative Results

#### Do PCIT Therapists Prefer the DPICS or the ECBI?

Qualitative analyses converged with the finding that therapists have more positive attitudes towards the DPICS than the ECBI (participant IDs follow quotes): “to be able to observe in person with the DPICS is so powerful” (9). Though attitudes were higher for the DPICS than the ECBI, themes also highlighted characteristics that were seen as positive about both, which was consistent with the overall positive attitudes across the two measures. Overall, the main theme that emerged in therapist descriptions of their preferences related to the utility of both measures to inform case conceptualization and treatment, with the following subthemes highlighting: (1) the types of data offered by each measure type, (2) the perceived reliability of each measure type, (3) their ease of use, (4) their contributions to motivating clients, and (5) their contributions to guiding session content. Themes and subthemes are presented in Table 3 and elaborated below.

**Table 3:** Themes and illustrative quotes; DPICS and ECBI

DPICS	ECBI
1. Type of data	
Relationship: I can get a sense of the level of warmth, the level of interest, it feels like more depth than the ECBI, and there’s not the filter from the parent, like I can see it without their own, filtered through their own beliefs, or expectations. (2) Parent Skills: I do think it’s a good measure of progress over time, quantitively, to see how many of the PRIDE skills they’re using and then also qualitatively, just to look at the relationship and how it changes over time. (20) Interactions: I think using the DPICS is helpful because I get to observe the interaction between the caregivers and the children, and you kind of get to see where there are some challenges with the parents as far as if they’re able to communicate effectively with their child, like how harsh they can be, or how passive. I think it really clues us into parenting styles. I think also it can give us kind of a small glimpse into how the child is reacting to the parent (5)	Recent Behaviors: What I like about the ECBI is that it’s quick and it’s time limited, like it’s for the week so it’s a good way to track progress. Most other measures are not written like that, they’re not written as, like at least child measures, now that I’m thinking about it like, the Beck Depression Inventory, the Beck Anxiety, they’re meant to give weekly, but there really aren’t many child measures meant to give weekly. (8) Parent Perception: I truly use it anecdotally, bringing the parents in and showing them this score and really talking about what they feel has shifted to gain a better understanding. And so that’s often where I end up finding this information where dad says like, “I’m just noticing it more”. So I try and use it because I think it’s a good quantitative measure of the behavior, but then I also feel like it allows them to have a conversation that gives me a lot of fast qualitative data around both parents’ understanding of the child, and what they notice. (13)
2. Ease of use	
You need [the DPICS] for being able to measure parents’ use of skills and progress in treatment. I’ve also found it very helpful in being able to quantify change for parents and show them their progress as we work together. I also think it’s a really valuable training tool in teaching my students about more objective measures and observational measures of parent-child interactions (1) I find it very telling as far as letting me know how the family is doing during the week, if they’re able to practice the skills, if they’re understanding the skills, it kind of lets me know as far as the parent, what their interpretation is of the skills, how often they’re practicing and then even whether or not there is an improvement in the parent-child interaction. I can see the dynamic pretty quickly within the five minutes. (16)	Since I’m still somewhat new to the process, it’s harder for me to look at the ECBI and really quickly be able to, like I can score it quickly obviously, but like it’s more, I find the data that I get with the DPICS is just richer, and I just have a harder time synthesizing the ECBI, I mean obviously the number is helpful, but then like the line by line analysis I’m just not quick enough, I’m not familiar with it enough yet to have it be really useful (14) I try to do it most weeks, but for parents who are really slow, like just have a difficult time answering it and a difficult time with reading it takes a lot of time out of our session for me to read that to them every time. (18)
3. Perceived reliability	
The DPICS is definitely the most reliable and valid measure to me. In self-report measures there’s a lot of layers that often times sort of obfuscate actual information, it doesn’t feel like as much of a direct reflection of what’s happening as something like the DPICS. (14)	Sometimes I see families just circling really quickly- they’re not even really thinking about the child’s behavior throughout the week, they’re just thinking in that moment - how were they before they got to session. (16)
4. Motivation	
Actionable skills measure: I’ll show them these were your skills last week. So I’ll really go through and they can see what their improvement is, and I found that’s really helpful, especially for parents that are like, “When are we gonna get to time-outs?” or “When are we gonna get to like telling him what not to do?”. I found it really helpful in saying to them, “This is what you need to meet mastery, and you’re doing so well, and here are some things that we can work on.” Just so they can see, I’m doing it right, but also here’s where the question piece is an issue, so I find it really helpful to do it every week, because we’re really tracking progress, and I think it recommits the family every week, to say like, “Oh this is why we have to do this.” (13)	Empowering: I think that clients see that, not only do they see the change in treatment, not only do they think treatment is effective, but they also feel that their therapist is more invested in their treatment. Their motivation sometimes is a little bit, you know, not so great in the beginning, um, but with PCIT and with the use of measures it really makes it more of a collaborative experience. You know, they’re empowered because they’re giving you information and that information is crucial to your work with the family, so you really depend on the client and the client also depends on you. (3) [Parents] love it. It really helps to keep parents motivated because they can see clearly on paper, and they’re the ones that are giving the scores each week, so it helps keep parents motivated, and it puts things into perspective when it shows how things are improving, things are getting better. You know often times they don’t realize that and the ECBI really helps to show that over time. (6)
5. Guiding sessions	
Definitely helps me focus on what I’m looking at in terms of the parent skills. it helps me structure my feedback and my coaching, and my guidance, helps me with consistency. (12)	It is really helpful because it’s going to guide my weekly session, so if a parent indicated that the child is having a hard time sitting still, for example, then maybe in that particular session I will focus a lot of my coaching around helping the parent describe so that the child can stay focused and seated, or praising the child for being able to stay seated and focused, so it’s going to guide the session depending on what items the parent endorsed were an issue. (17)

##### Type of Data Impacts Clinician Preference

Therapists consistently referenced the important lens the DPICS offered them into the nature and quality of the parent–child interaction. Therapists described the quality and types of information they get from the DPICS:

The DPICS I find is helpful to see, especially in two parent or multi-caregiver households, how the child responds differently to each caregiver. That’s more of a qualitative assessment, but that’s what’s most helpful because caregivers have a hard time describing that so it’s helpful to observe. Very few caregivers have skills so the coding confirms what is assumed, so it’s good to confirm that quantitatively but I find qualitatively there’s a lot of good information on differential responding of the child between caregivers. (4)

Interestingly, much of the information that therapists described as being helpfully provided by the ECBI involved understanding the parents. The ECBI is a report of both child behaviors and how much those behaviors troubles the parents. As described by one therapist:

I clinically find it useful to know if there's a discrepancy between the intensity and the problem scores because if it’s super low intensity and high problem then that’s going to affect how I work with the parent. Those are parents I know need more realistic understanding of child development and expectations, and probably need a lot more coaching on coping skills and also just their own stress levels, those parents I’m gonna be a lot more in tune with what are they doing for self-care each week, do they have supports, things like that. (11)

##### Perceived Reliability Impacts Clinician Preference

Therapists expressed that the immediacy of the data made available by the DPICS felt more credible to them than parent symptoms reports, “I really like the DPICS because of the fact that it is an observational measure and more of an objective rating of observed behavior which you don’t always get from self-report rating measures” (1).

They described that although the ECBI provided helpful information, they often questioned its accuracy, while the live behavioral observation “[felt] like it’s a lot more telling and true to what’s going on” (16). Therapists mentioned that parent stress, negative self-appraisals, and other factors contributing to parent perceptions may contribute to the inaccuracy of parent-reports:

I find like with the parent reports you get good information, but there’s an element of the parent’s perception sort of coloring how they’re reporting things. So, a really overwhelmed parent who might not be doing so bad might report themselves as doing worse than they actually are. And if you’re able to observe them directly with something like the DPICS, I think it gives you sometimes a more realistic, um, measure of what’s happening. (3)

##### Ease of Use Impacts Clinician Preference

Clinicians discussed the practicality of both the ECBI and the DPICS. Although some voiced appreciation for the brevity of the ECBI (“it’s a quick measure—I actually really like the ECBI” [8]), many others described it as much too time-consuming, particularly for families with literacy or language issues:

Thirty or 40% of the Spanish speakers on my caseload don’t read well enough to fill it out. And then doing it orally is extremely time consuming, just because it is to read it out loud and also because when you’re doing a measure orally people tend to think it’s more of a conversation. Especially if they’re not used to standardized measures in the first place, and so it can take 30 minutes sometimes just to fill out the ECBI. (19)

Another therapist described needing to skip the weekly parent-report measure due to how much time it took for some families to complete:

When that is a challenge in the parents that is completing the ECBI and I have to do it with them, it is definitely a barrier because it takes over the session. So in those cases I don't do it weekly, I might do it eh, twice a month eh, or maybe once a month in some cases. (2)

##### Measures Help Motivate Families

Clinicians frequently discussed the clinical utility of the measures in their ability to increase caregiver motivation for treatment. They felt that the measures “can be very empowering” (3), providing confirmation for both the therapist and family that treatment is working:

[The ECBI] is awesome feedback for us; it’s very motivating as a PCIT therapist to see - it’s very validating to see like okay, this treatment is working where these parents are starting to see changes, even if they’re not huge changes—we can show them “Look! You are slowly starting to rank these things lower and lower as the weeks go on” So, I love it. (22)

Clinicians described the DPICS as being particularly motivating since it measures actionable parenting skills, and provides clear scores and goals that parents can work towards. It is more difficult to see the direct connection between scores on symptom reports and parenting behaviors, while the DPICS, which measures parenting skill use, provides immediate feedback to parents on things they can work towards:

I find [the DPICS] very helpful to be able to give parents a regular measurement of their progress, and I think that parents typically respond very well to having that type of feedback about progress. I have found it extremely beneficial and especially working through some very resistant parents, especially those who have been court ordered to come to the counseling. (15)

##### Measures Help Guide Treatment

Many therapists described how both types of measures help with treatment planning, including providing guidance for a session: the DPICS “really informs the rest of my session” (21), and “[the ECBI] helps me when I see … this week you struggled with this area, and then we’ll focus on some coaching around that” (23). They indicated that in addition to offering them a roadmap for session, using measures and sharing results helped parents trust their coaching and session content:

The parents I think trust more of the process because you’re showing them, “okay, here’s where you were during the five minutes, here’s what we’ll work on because these two things were low”, and at the end of the session giving that feedback. (11)

Therapists also reported that the ECBI helped ensure that they targeted behaviors that the parents reported were particularly problematic: “I love [the ECBI] because it really helps us to see what behaviors are extremely problematic for our parents, and we can really fine-tune our coaching to really address some of those problem behaviors” (22). Interestingly, although therapists often questioned the accuracy of parent reports, as seen in the “perceived reliability” theme, they also realized the importance of meeting parents where they are and making treatment responsive to parental concerns.

#### Do PCIT Therapists Prefer Measures used within PCIT or those used in General Practice?

Qualitative analyses converged with findings that participants rated PCIT progress measures as higher than the perceived practicality of progress measures in general practice and illuminated reasons for this preference. Themes that emerged related to this expressed preference included (1) integration of measures into the treatment modality and (2) unique combination of measure type in PCIT. Illustrative quotes are presented in Table 4 and themes are described further below.

**Table 4:** Themes and illustrative quotes; PCIT and general practice

PCIT	General practice
1. Integration of measures in modality	
It’s really cool to see the changes week to week with PCIT clients. I mean everything that you’re doing in PCIT is so structured, is so specific, and I think it’s really helpful for people to be able to see that change in something so intangible, is what therapy is usually thought to be. I’ve wanted to sort of recreate that for my clients who are not in PCIT so they can have a similar experience. I think it’s a fine line though, between trying to use the measures to inform treatment and really, you know, trying to keep it simple for your clients so they can actually do them without feeling too overwhelmed. (3)	Outside of PCIT, I probably wasn’t giving them as consistently or I didn’t really know how to use them to inform treatment planning and I think that PCIT taught me a lot about how to sit down and look at data with parents. Um, and I think that now, now it’s just is easier for me to do and more automatic. (20)
	The ECBI is weekly typically for PCIT clients, and then for non-PCIT clients, there’s not really any weekly measure that will show us week by week how things are progressing or deteriorating. So with non-PCIT clients, they get less frequent assessment and monitoring. a lot of it is just monitoring the client in session, like based on clinical judgement observation and talking to the client, talking to the parent, so it’s not necessarily like a standardized way for non-PCIT clients (5)
[progress monitoring in PCIT] is so much more structured, and it’s much more of the routine. I think that’s why I like PCIT so much to be honest and TFCBT too is just, TFCBT tends to be heavier and with the trauma piece too, but I think because it is so measured that’s why, part of why I like providing it. (9)	
2. Combination of measure types	
With PCIT families, in the first two sessions from the measures and from the DPICS, I’ve gotten a lot of information, whereas with my other families have been a lot more interviews, filling out the measures, waiting for those to come back to me, I’m engaging the child in some sort of play, to structure it, to get more observation, uhm, so I have to get a little bit more creative, which is why I love PCIT because of its structure and it gives me all the tools that I need. (16)	[outside of PCIT] with younger children I tend to do a behavioral observation as well, and it would be like a, like a CDI sort of type session. Um, that’s more, you know more loosely related to PCIT, just to see what happens. (3)
	I’ll do a DPICS even on a case that I have a feeling is not a fit for PCIT, at least initially, just because I want to see what that interaction looks like. (13)
I love comparing my observations in DPICS to how their ECBI scores are. (18)	

##### Integration of Measures into Treatment Modality

One of the primary differences therapists described between measures used in PCIT and standardized measures in general practice included how they are embedded in the former: “you can’t do PCIT without using the DPICS” (1). As a result, there is a clear protocol guiding both when to administer them and how to use them to guide treatment and motivate families, whereas in general practices “that objective data doesn’t guide my session” (11). While therapists described working in agencies with measure requirements, they voiced more frustration with these requirements, despite them being less frequent than PCIT measure requirements, suggesting that they perceived progress monitoring outside of PCIT to be more burdensome.

After seeing the value of [using measures] in PCIT and how it can drive treatment in a session I have more appreciation for the use of data, however having been a frontline clinician in a community mental health agency for so long, I know that it’s near impossible to do that. It was very difficult even in a PCIT session to do it. There is excessive paperwork already in those environments, because of all of the accrediting bodies and regulations, documentation takes much longer because of these things. If you’re also throwing this other measure out there, it’s a lot when you can just do treatment as usual, right? (11)

Despite this frustration with the additional paperwork associated with progress measures, many of the interviewees described an appreciation for the higher rate of progress monitoring in PCIT: “I like the more frequent monitoring with PCIT” (14). They felt like they knew how to use the measures within each session, and had clear ways of sharing feedback with families within each session. In contrast, they reported being unsure how to use measures to inform and guide treatment in general practice, and as a result using them less frequently: “most of the time, if you’re not doing PCIT and it’s not kind of part of your programming, or your – like your clinical makeup – then you’re not really using the measure” (16).

Clinicians often described less frequent use of measures in general practice (once monthly, once every six months, or just pre- and post-treatment). Although the ASA-MF score for standardized assessments in general practice among this sample was still positive, despite being less positive than attitudes towards PCIT-specific measures, clinicians simply reported not being inclined to use measures if they were not indicated in treatment protocol:

We don’t have any protocols around specific measures to use in between, although staff have access to a battery of assessments that they could use if they want. Anecdotally speaking, staff here, including myself, tend to not use measures in between because we sort of get in the flow of just seeing clients week to week and bringing them into therapy, and it just doesn’t come into my consciousness as much as it does for PCIT. (17)

##### Combination of the Measure Types

This theme is related to therapist preferences for behavioral observation, as many therapists interviewed suggested that the information they gathered from the two types of measures informed one another. The measures most interviewees discussed using in general practice were paper and pencil measures, though a few mentioned integrating the DPICS or other behavioral observations into their general practices because they found them so helpful. Therapists described appreciating the combination of data offered by the two types of measures, and finding the clinical picture more comprehensive as a result of integrating the behavior observations with parent-report, both in session and during the intake: “I’m trying to do the DPICS really quickly after, so that I’m sort of conceptualizing very quickly, is what I’m observing also lining up with the parent report” (13).

The speedy gathering of this rich information stood in contrast for some clinicians to the slower and more paperwork-heavy intake assessments required in their agencies or in practices that did not include a behavioral observation measure. In contrast to the quote above, which highlights the speed at which a comprehensive picture is created from the integration of two measure types, therapists described a slow and burdensome assessment process in general practice:

What stops me is that ideally I would have all of the measures be completed at the same time and then they’re all scored at the same time, and then I can give feedback, but usually I’m waiting to get another one, like, they did the TSCC and I’m still waiting for the UCLA, and then by the time they’re bringing it back, it’s two weeks later, and so I have like all these measures that are like somewhat different data points, because they were done at different times. And then by that time, I’m like oh yeah, I guess I should give them feedback, it’s kind of outdated at this point, but usually I’ll give, like if there’s something that I notice that seems really significant, then I’ll remember to do it, but if there’s nothing that’s super significant, or if I doubt the validity of their measures, then I oftentimes don’t. (14)

## Discussion

It is important to understand therapist preferences and attitudes when attempting to implement evidence-based quality improvement tools, such as progress measures, in community mental health (Aarons, 2004; Aarons, et al., 2011; Jensen-Doss et al., 2020; Sale et al., 2020). Investigating the attitudes of therapists who already demonstrate higher use of measures and more experience with them due to being trained in an assessment-driven protocol provides helpful insight into what factors may impact therapist preference. The findings from this study indicated that PCIT therapists prefer the DPICS to the ECBI and prefer measures embedded in PCIT to general standardized assessment. Therapists in this study had positive attitudes towards the practicality of both the DPICS and ECBI, with slightly higher attitudes towards the DPICS. Their preferences were shaped by the type of information they gathered from each measure, how reliable they found the measure, how easy the measure was to implement (how quick and accessible), and the ways in which it could be used clinically, to motivate parents or to guide session content.

Therapists in this study appreciated the combination of two different types of measures, since the DPICS is a behavioral observation measure while the ECBI is a parent report measure. They expressed that each measure offers distinct types of information. The DPICS offers insight into the quality of the caregiver-child relationship, parenting skills, and caregiver behaviors, while the ECBI offers a lens into caregiver perceptions, stress levels, buy-in, and discrepant perspectives between caregivers. Previous research indicates that of the types of information clinicians want from progress measures, family functioning and quality of parent-youth relationship ranked among the highest, which the current findings substantiate (Bickman et al, 2000).

In combination, the DPICS and ECBI give therapists a more comprehensive clinical picture. These results corroborate previous suggestions that measuring multiple dimensions of change is important (Smith & Jensen-Doss, 2017). They also suggest that measures can function beyond their intended use. For example, the ECBI presents itself as a measure of problematic child behaviors, however therapists extracted much more information from it (Eyberg et al., 1999). The functions of these measures also extended beyond simply monitoring symptoms and skills, to motivating families and guiding individual sessions. Therapists seemed to value PCIT measures, which were embedded in the treatment protocol and served multiple of these functions, over measures in general practice, which do not have as many immediately evident uses.

While previous research has suggested that practicality may be the primary determinant of therapist’s attitudes towards and use of measures (Jensen-Doss & Hawley, 2010; Jensen-Doss et al., 2018a, 2018b), this study provides some indication about what features clinicians find to be practical. The DPICS is an involved measure, requiring extensive familiarity with a complex coding system and therapist engagement for either 20 min per caregiver during pre- and post- treatment sessions or five minutes per caregiver during typical sessions (Eyberg et al., 2013; Eyberg & Funderburk, 2011). Given the extensive training needed for the DPICS, it was unexpected that participants rated it as more practical. Qualitative results in this study suggested that high practicality ratings were contingent upon practical elements of the DPICS, including consistent timing (it typically takes 5 min during sessions), and the immediacy of results and feedback. While time required to complete other measures depend upon client variables, such as caregiver literacy, the timing of the DPICS is more predictable.

The current study compared scores on the ASA-MF practicality subscale because in the past it has been identified as the only predictor of measure use (Jensen-Doss & Hawley, 2010; Jensen-Doss et al., 2018a, 2018b). However, qualitative data analysis in the current study indicates that practicality can be conceived of in multiple ways. High training needs, complex coding systems, and therapist time commitment during session are practical elements that ostensibly render the DPICS less practical than paper-and-pencil measures that can simply be handed out before session and collected. However, immediacy of scoring, ability to provide instant feedback, and embeddedness in session were practical elements that boosted therapist preference for this unique measure. Additionally, the measure is given almost every week, so frequency or time commitment did not seem to detract from therapist preference. Consideration of additional elements impacting a measure’s practicality should be considered in the future, as they appear to impact clinician attitudes towards progress measures.

This study additionally found that identifying as Hispanic/Latinx as compared to Non-Hispanic white significantly predicted less positive attitudes towards measures. A previous study of perceptions of progress monitoring among ethnic minority community therapists who were 69.9% Hispanic found that therapist cultural identity (affiliation to culture of origin) predicted less positive attitudes on the ASA-MF (Rodriguez et al., 2019). The present study did not include a measure of cultural identity, but further research should investigate attitudes of ethnic minority therapists towards measures. Although it did not emerge as a preliminary theme in our qualitative data analysis, therapists referenced challenges using paper-and-pencil measures with families with lower literacy rates or ethnic minority families for whom certain concepts such as “dawdling” may not be culturally meaningful. It is possible that Latinx and Hispanic therapists may be more sensitive to issues of cultural incongruence between certain measures and their clients’ beliefs, which could drive their lower ratings. Further efforts are needed to address how progress monitoring can be implemented with health equity and cultural congruence in mind (Liu et al., 2019).

## Limitations

Although this study offers insight into therapist preferences as they relate to types and context of progress measures, it had several limitations, which should frame interpretation of its findings. The sample comprised therapists trained in PCIT who share many attributes, which may impact their preferences and attitudes or pre-incline them to more positive overall attitudes than therapists at large. This sample served the purposes of this study, enabling us to compare types of measures that we knew therapists had familiarity with, and to consider measures used both in and outside of a specific protocol. However, these results may not generalize to all community-based therapists who have not received training in particular evidence-based protocols. In order to gain further insight into the attitudes of community-based therapists towards types of measures, research should include a wider sample of providers.

Additionally, this study did not evaluate clinician preferences for specific types of measures in general practice. Although the primary quantitative outcome measures gathered information about preferences for two distinct types of measures in PCIT, the type of standardized progress measures used in general practice was not specified. Conclusions about preference towards measure type in general practice cannot be drawn, and preferences towards specific measures in PCIT may be specific to PCIT. Although therapists preferred the DPICS in the context of PCIT, this may not generalize to treatment as usual or other therapeutic practices.

This study also looked specifically at therapist perceptions of measures, and did not consider how caregiver or family preferences may shape provider preferences. It is possible that therapists have more positive attitudes towards measures that parents also find useful, and attitudes expressed by providers may actually reflect satisfaction by everyone involved in treatment, given the many functions that measures serve in PCIT. In the future, it may be helpful to disentangle therapist and caregiver attitudes and to assess the extent to which they are related.

## Implications and Future Directions

Despite its limitations, this study provides insight into therapist preferences for certain measures over others. Since therapist attitudes are pivotal points of investigation and shape the services clients receive, they must be examined when considering how to increase the implementation of a quality-improvement tool. This study demonstrates that training may shape therapist attitudes towards measures, and that receiving training in an assessment-driven protocol likely improves these attitudes. Additionally, it offers a helpful perspective on the ongoing endeavor to understand low use of measures in mental health care, suggesting that many therapists greatly appreciate measures, but prefer particular types and combinations of measures, and prefer measures in certain treatment modalities. In order to increase therapist measure use, future implementation research and efforts should be responsive to therapist preferences as highlighted here, increasing the dissemination of those specific measures that therapists find valuable. Mental health treatment has much to gain from attending to therapist preferences and offering them the tools that they find most practical.

## Data Availability

The interviews generated and analyzed during the current study are not publicly available to protect the identities of participants, but are available from the corresponding author on reasonable request.

## References

[CR1] Aarons GA (2004). Mental health provider attitudes toward adoption of evidence-based Practice: The Evidence-Based Practice Attitude Scale (EBPAS). Mental Health Services Research.

[CR2] Aarons GA (2005). Measuring provider attitudes toward evidence-based practice: Consideration of organizational context and individual differences. Child and Adolescent Psychiatric Clinics of North America.

[CR3] Aarons GA, Hurlburt M, Horwitz SMC (2011). Advancing a conceptual model of evidence-based practice implementation in public service sectors. Administration and Policy in Mental Health and Mental Health Services Research.

[CR4] American Psychological Association (2006). Evidence-based practice in psychology. American Psychologist.

[CR5] Bahl A, Spaulding S, McNeil C (1999). Treatment of noncompliance using Parent Child Interaction Therapy: A data-driven approach. Education and Treatment of Children.

[CR6] Barnett M, Brookman-Frazee L, Regan J, Saifan D, Stadnick N, Lau A (2017). How intervention and implementation characteristics relate to community therapists’ attitudes toward evidence-based practices: A mixed methods study. Administration and Policy in Mental Health and Mental Health Services Research.

[CR7] Beidas RS, Marcus S, Aarons GA, Hoagwood KE, Schoenwald S, Evans AC, Hurford MO, Hadley T, Barg FK, Walsh LM, Adams DR, Mandell DS (2015). Predictors of community therapists’ use of therapy techniques in a large public mental health system. JAMA Pediatrics.

[CR8] Beveridge RM, Fowles TR, Masse JJ, McGoron L, Smith MA, Parrish BP, Circo G, Widdoes N (2015). State-wide dissemination and implementation of parent–child interaction therapy (PCIT): Application of theory. Children and Youth Services Review.

[CR9] Bickman L, Kelley SD, Breda C, de Andrade AR, Riemer M (2011). Effects of routine feedback to clinicians on mental health outcomes of youths: Results of a randomized trial. Psychiatric Services.

[CR10] Bickman L, Lyon AR, Wolpert M (2016). Achieving precision mental health through effective assessment, monitoring, and feedback processes. Administration and Policy in Mental Health and Mental Health Services Research.

[CR11] Bickman L, Rosof-Williams J, Salzer MS, Summerfelt WT, Noser K, Wilson SJ, Karver MS (2000). What information do clinicians value for monitoring adolescent client progress and outcomes?. Professional Psychology: Research and Practice.

[CR12] Boswell JF, Kraus DR, Miller SD, Lambert MJ (2015). Implementing routine outcome monitoring in clinical practice: Benefits, challenges, and solutions. Psychotherapy Research.

[CR13] Braun V, Clarke V (2006). Using thematic analysis in psychology. Qualitative Research in Psychology.

[CR14] Brookman-Frazee L, Drahota A, Stadnick N (2012). Training community mental health therapists to deliver a package of evidence-based practice strategies for school-age children with autism spectrum disorders: A pilot study. Journal of Autism and Developmental Disorders.

[CR15] Damschroder LJ, Aron DC, Keith RE, Kirsh SR, Alexander JA, Lowery JC (2009). Fostering implementation of health services research findings into practice: A consolidated framework for advancing implementation science. Implementation Science.

[CR16] Edbrooke-Childs J, Barry D, Rodriguez IM, Papageorgiou D, Wolpert M, Schulz J (2017). Patient reported outcome measures in child and adolescent mental health services: Associations between clinician demographic characteristics, attitudes and efficacy. Child and Adolescent Mental Health.

[CR17] Ehrenreich-May J, Southam-Gerow MA, Hourigan SE, Wright LR, Pincus DB, Weisz JR (2011). Characteristics of anxious and depressed youth seen in two different clinical contexts. Administration and Policy in Mental Health and Mental Health Services Research.

[CR18] Eyberg SM, Boggs S, Jaccard J (2014). Does maintenance treatment matter?. Journal of Abnormal Child Psychology.

[CR19] Eyberg, S. M., Nelson, M. M., Ginn, N. C., Bhuiyan, N. & Boggs, S. R. (2013). *Dyadic Parent-Child Interaction Coding System-Fourth Edition*. PCIT International, Inc

[CR20] Eyberg, S. M., & Pincus, D. (1999). *Eyberg Child Behavior Inventory and Sutter-Eyberg Student Behavior Inventory-revised professional manual.* Psychological Assessment Resources

[CR21] Eyberg, S. M. & Funderburk, B. (2011). *Parent-child interaction therapy: Treatment manual.* PCIT International

[CR22] Garland AF, Kruse M, Aarons GA (2003). Clinicians and outcome measurement: What’s the use?. The Journal of Behavioral Health Services & Research.

[CR23] Goodyear RK, Wampold BE, Tracey TJG, Lichtenberg JW (2017). Psychotherapy expertise should mean superior outcomes and demonstrable improvement over time. The Counseling Psychologist.

[CR24] Hatfield D, McCullough L, Frantz SHB, Krieger K (2009). Do we know when our clients get worse? an investigation of therapists’ ability to detect negative client change. Clinical Psychology & Psychotherapy.

[CR25] Hennink MM, Kaiser BN, Marconi VC (2017). Code saturation versus meaning saturation: How many interviews are enough?. Qualitative Health Research.

[CR26] Jakobsen JC, Gluud C, Wetterslev J, Winkel P (2017). When and how should multiple imputation be used for handling missing data in randomised clinical trials–a practical guide with flowcharts. BMC Medical Research Methodology.

[CR27] Jensen-Doss A, Douglas S, Phillips DA, Gencdur O, Zalman A, Gomez NE (2020). Measurement-based care as a practice improvement tool: Clinical and organizational applications in youth mental health. Evidence-Based Practice in Child and Adolescent Mental Health.

[CR28] Jensen-Doss A, Haimes EMB, Smith AM, Lyon AR, Lewis CC, Stanick CF, Hawley KM (2018). Monitoring treatment progress and providing feedback is viewed favorably but rarely used in practice. Administration and Policy in Mental Health and Mental Health Services Research.

[CR29] Jensen-Doss A, Hawley KM (2010). Understanding barriers to evidence-based assessment: Clinician attitudes toward standardized assessment tools. Journal of Clinical Child & Adolescent Psychology.

[CR30] Jensen-Doss A, Smith AM, Becker-Haimes EM, Mora Ringle V, Walsh LM, Nanda M, Walsh SL, Maxwell CA, Lyon AR (2018). Individualized progress measures are more acceptable to clinicians than standardized measures: Results of a national survey. Administration and Policy in Mental Health and Mental Health Services Research.

[CR31] Lambert MJ, Shimokawa K (2011). Collecting client feedback. Psychotherapy.

[CR32] Lieneman CC, Brabson LA, Highlander A, Wallace NM, McNeil CB (2017). Parent-Child Interaction Therapy: Current perspectives. Psychology Research and Behavior Managemen.

[CR33] Little RJ (1988). A test of missing completely at random for multi-variate data with missing values. Journal of the American Statistical Association.

[CR34] Liu FF, Cruz RA, Rockhill CM, Lyon AR (2019). Mind the gap: Considering disparities in implementing measurement-based care. Journal of the American Academy of Child and Adolescent Psychiatry.

[CR35] Lyon AR, Stanick C, Pullmann MD (2018). Toward high-fidelity treatment as usual: Evidence-based intervention structures to improve usual care psychotherapy. Clinical Psychology: Science and Practice.

[CR36] Merikangas KR, He J, Burstein M, Swanson SA, Avenevoli S, Cui L, Benjet C, Georgiades K, Swendsen J (2010). Lifetime prevalence of mental disorders in U.S. adolescents: Results from the national comorbidity survey replication–Adolescent supplement (NCS-A). Journal of the American Academy of Child & Adolescent Psychiatry.

[CR37] Nelson TD, Steele RG, Mize JA (2006). Practitioner attitudes toward evidence-based practice: Themes and challenges. Administration and Policy in Mental Health and Mental Health Services Research.

[CR38] Palinkas LA, Aarons GA, Horwitz S, Chamberlain P, Hurlburt M, Landsverk J (2011). Mixed method designs in implementation research. Administration and Policy in Mental Health and Mental Health Services Research.

[CR39] Palinkas LA, Horwitz SM, Green CA, Wisdom JP, Duan N, Hoagwood K (2015). Purposeful sampling for qualitative data collection and analysis in mixed method implementation research. Administration and Policy in Mental Health and Mental Health Services Research.

[CR40] Park AL, Tsai KH, Guan K, Chorpita BF (2018). Unintended consequences of evidence-based treatment policy reform: Is implementation the goal or the strategy for higher quality care?. Administration and Policy in Mental Health and Mental Health Services Research.

[CR41] Reding MEJ, Chorpita BF, Lau AS, Innes-Gomberg D (2014). Providers’ attitudes toward evidence-based practices: Is it just about providers, or do practices matter, too?. Administration and Policy in Mental Health and Mental Health Services Research.

[CR42] Rivenbark JG, Odgers CL, Caspi A, Harrington H, Hogan S, Houts RM, Poulton R, Moffitt TE (2018). The high societal costs of childhood conduct problems: Evidence from administrative records up to age 38 in a longitudinal birth cohort. Journal of Child Psychology and Psychiatry.

[CR43] Rodriguez A, Lau AS, Wright B, Regan J, Brookman-Frazee L (2018). Mixed-method analysis of program leader perspectives on the sustainment of multiple child evidence-based practices in a system-driven implementation. Implementation Science.

[CR44] Rodriguez A, Terrones L, Brookman-Frazee L, Regan J, Smith A, Lau AS (2019). Associations between cultural identity and attitudes toward routine progress monitoring in a sample of ethnically diverse community therapists. Psychological Services.

[CR45] Rye M, Rognmo K, Aarons GA, Skre I (2019). Attitudes towards the use of routine outcome monitoring of psychological therapies among mental health providers: The EBPAS–ROM. Administration and Policy in Mental Health and Mental Health Services Research.

[CR46] Sale R, Bearman SK, Woo R, Baker N (2020). Introducing a measurement feedback system for youth mental health: Predictors and impact of implementation in a community agency. Administration and Policy in Mental Health and Mental Health Services Research.

[CR47] Smith AM, Jensen-Doss A (2017). Youth psychotherapy outcomes in usual care and predictors of outcome group membership. Psychological Services.

[CR48] Solstad SM, Castonguay LG, Moltu C (2019). Patients’ experiences with routine outcome monitoring and clinical feedback systems: A systematic review and synthesis of qualitative empirical literature. Psychotherapy Research.

[CR49] Southam-Gerow MA, Rodríguez A, Chorpita BF, Daleiden EL (2012). Dissemination and implementation of evidence based treatments for youth: Challenges and recommendations. Professional Psychology: Research and Practice.

[CR50] Spradley J (1979). The ethnographic interview.

[CR51] Timmer SG, Urquiza AJ, Boys DK, Forte LA, Quick-Abdullah D, Chan S, Gould W (2016). Filling potholes on the implementation highway: Evaluating the implementation of Parent-Child Interaction Therapy in Los Angeles County. Child Abuse and Neglect.

[CR52] Warren JS, Nelson PL, Mondragon SA, Baldwin SA, Burlingame GM (2010). Youth psychotherapy change trajectories and outcomes in usual care: Community mental health versus managed care settings. Journal of Consulting and Clinical Psychology.

